# A Spatial Structure of Key Tree Species *Metrodorea nigra* St. Hill. (Rutaceae) Is Associated with Historical Disturbance and Isolation in Southeastern Brazil

**DOI:** 10.3390/plants14050702

**Published:** 2025-02-25

**Authors:** Rômulo Maciel de Moraes Filho, Fernando Bonifácio-Anacleto, Fabio Alberto Alzate-Martinez, Carlos Alberto Martinez, Ana Lilia Alzate-Marin

**Affiliations:** 1 Plant Genetics Laboratory, Department of Genetics, Faculty of Medicine of Ribeirão Preto (FMRP-USP/RP), University of São Paulo, Ribeirão Preto 14049-900, Brazil; bonifacioanacleto@usp.br; 2 Graduate Program, Department of Genetics, Faculty of Medicine of Ribeirão Preto (FMRP-USP/RP), University of São Paulo, Av. Bandeirantes 3900, Ribeirão Preto 14049-900, Brazil; 3 Nawi Spatial Design and Research, Ribeirão Preto 14040-160, Brazil; nawistudio.contact@gmail.com; 4 Department of Biology, Ribeirão Preto School of Philosophy, Science and Literature (FFCLRP), University of São Paulo, Av. Bandeirantes 3900, Ribeirão Preto 14040-901, Brazil; carlosamh@ffclrp.usp.br

**Keywords:** *Metrodorea nigra*, inbreeding, spatial genetic structure (SGS), anthropogenic disturbances

## Abstract

The semi-deciduous Brazilian Atlantic Forest has faced intense fragmentation, impacting *Metrodorea nigra* St. Hill., a fly-pollinated and autochorous tree. We investigated population structure, inbreeding, and spatial genetic structure (SGS) across adult (Adu) and juvenile (Juv) generations in three fragmented populations of *M. nigra* in Ribeirão Preto, São Paulo, Brazil. We tested whether the magnitude of these effects could result from its mating system, seed dispersal, anthropogenic disturbances, matrix, and fragment size. Populations affected by selective logging, fire, and trail openings include M13-Rib (84 ha) and FAC-Crav (8 ha), both surrounded by sugar cane and BSQ-Rib (3 ha) in an urban matrix. We evaluated phenological events and germination rates in the BSQ-Rib fragment. We sampled leaves and amplified their DNA using ISSR (UBC 1, 2, 820, 834, 851, 858, 860, 886) and SSR (Mtn 1, 3, 13, 16, 19, 87, 95) molecular markers. Fst, PCoA, and AMOVA values suggest a lack of generational isolation, with most variance within generations. Inbreeding values were significant in all populations (Fis and Fit, *p* = 0.001), probably intensified by natural seed dispersal and pollinator behavior favoring geitonogamy. However, fragmentation, anthropogenic disturbances, and the surrounding matrix influenced SGS. The urban BSQ-Rib fragment recorded the highest SGS values (26 m Juv, 24 m Adu [ISSR]; 7 m Juv, 9 m Adu [SSR]), which may result in low fruit and seed production and germination rates. Despite being the largest fragment, M13-Rib shows SGS in the first distance class (19 m Juv, 24 m Adu [ISSR]; 0 m Juv, and 10 m Adu [SSR]), possibly due to selective logging and fire. FAC-Crav, a more conserved fragment, showed no SGS in adults but punctual SGS in juveniles (27 m [ISSR] and 8 m [SSR]), pointing to it as a promising source for seed collections for reforestation purposes. In summary, inbreeding in *M. nigra*, influenced by pollinator behavior and seed dispersal, along with fragmentation, anthropogenic disturbances, and the surrounding matrix, are critical in shaping SGS. These factors potentially impact the reproductive success of *M. nigra* and their long-term survival in the face of climate change.

## 1. Introduction

In natural populations, plants are rarely randomly distributed and are often structured into gradients, aggregates, or other forms of spatial organization [1]. Because they are sessile organisms, plants have limited gene flow capacity, the primary determining factor for spatial genetic structure (SGS) [2,3,4]. SGS refers to the non-random distribution of genotypes in a two-dimensional space, leading to clusters of genetically related individuals [5,6,7,8,9]. SGS can be influenced by biotic factors, like limited pollen and seed dispersal, and abiotic factors, such as selective cutting and local extinction. On a smaller spatial scale, the formation of local neighborhoods due to limited gene flow is the most prevalent cause.

Plant phenology, defined as the observable patterns of flowering, fruiting, leaf fall, and budding events throughout the year, is directly influenced by climatic conditions and soil characteristics. These conditions determine all vegetative and reproductive patterns of plants and the animals (both vertebrate and invertebrate) that depend on them [10,11,12,13]. In higher plants, gene flow occurs primarily through the diploid embryos (seed flow) and the haploid male gametes (pollen flow). When pollinated and dispersed by the wind, abiotic factors are the prominent pollen and seed vectors. Other species depend on ecological relationships with animals for pollination and dispersal processes [14]. Forest species respond differently to the conditions to which they are subjected. Isolated fragments are more severely affected by genetic drift, the founder effect, and genetic erosion [15]. Thus, the level of gene flow between forest fragments and subpopulations is directly linked to the physical distance separating populations and the range of pollen and seed-dispersing agents. Restricted seed or pollen movement in outcrossing species may represent inbreeding due to the consanguinity of adjacent individuals or mating among relatives (multiple references in Loveless and Hamrick [2]), influencing SGS [2,3,4,5,16]. Additionally, many angiosperm flowers minimize self-pollination through temporal and spatial separation of pollen and stigma. However, these adaptations do not prevent pollen transfer between flowers on the same plant, known as geitonogamy [17].

The Brazilian Atlantic Forest has suffered intense fragmentation over the last centuries [18,19], especially in forests located on arable land, such as the semi-deciduous seasonal forest of the Ribeirão Preto region in the interior of São Paulo State, Brazil [20,21]. Studies on the spatial genetic structure of key species populations in these remnants are essential for understanding and conserving the remaining biological diversity, as well as providing a basis for implementing recovery measures for degraded flora [19,22]. In this context, the species *Metrodorea nigra* St. Hill. is associated with the presence of large tree species, as it is adapted to the understory and shading conditions of the semi-deciduous forests of the Brazilian Atlantic Forest [23,24,25,26,27,28,29]. In Ribeirão Preto, this species occurs in 19 semi-deciduous mesophilic forest fragments (75%) [20].

*Metrodorea nigra* is a monoecious evergreen tree species belonging to the Rutaceae family, popularly known as “carrapateira” or “caputuna-preta”. It is a typical sub-canopy species of the semi-deciduous forests of the Paraná River basin and the Brazilian Atlantic Forest, which can reach up to five meters in height [30]. The species, with an irregular and discontinuous distribution, occurs at a high density ranging between 394 and 500 individuals per hectare from Bahia to Paraná, and its wood is often used in construction [23,26,31,32,33]. *M. nigra* possesses self-incompatible hermaphrodite flowers, which are fly-pollinated by the species *Pseudoptilolepis nigripoda* Snyder (Muscidae) and *Fannia* spp. (Fanniidae), producing fruits in low quantities only through cross-fertilization [34]. The fruits, when dried, break and release the seeds explosively (autochory), with a low number of seeds produced annually [29,33,34]. In some forest fragments of the state of Paraná, monkeys consume immature seeds [33,35].

Since *M. nigra* develops preferentially in the most preserved regions of the woods, its presence indicates elevated conservation levels. Due to the high frequency and density at which *M. nigra* occurs, it has been classified as a species of great ecological importance [23,26,27,31], being considered a key species in forest nutrient dynamics [26]. Therefore, *M. nigra* could be used as an indicator species of forest fragment quality as its density may be affected by anthropogenic disturbances such as canopy opening and selective cutting [25,26,27]. In addition, the species is on the list of those recommended for ecological restoration in São Paulo State (Brazil), including seasonal semi-deciduous and dense ombrophilous forests, among others [36]. Detailed phenological research is essential to ensure that seeds can be effectively used in reforestation programs. Furthermore, to make decisions about sampling procedures that maintain maximum genetic diversity, it is also necessary to understand how genetic variation is distributed within a species. This requires knowledge (or estimation) of its genetic structure [2].

The *M. nigra* species has several interesting characteristics for genetic studies in the semi-deciduous seasonal forest of the Ribeirão Preto SP, Brazil region over time, such as (1) its wide occurrence in the shade of the most conserved areas in some remnants; (2) its size (4 to 5 m), which facilitates the collection of leaves and seeds; and (3) the existence of detailed studies on its genetic diversity, molecular phylogeny, biogeography, reproductive and anatomical biology, and pollinating and dispersing agents [30,34,35,36,37,38,39]. Previous studies by our group in this region, in three fragments of different sizes (M13-Rib—84 ha, FAC-Crav—8 ha, and BSQ-Rib—3 ha), showed high levels of genetic diversity, indicating that habitat reduction has little effect on the genetic variability of *M. nigra* [39]. Variation patterns studied with structure, principal coordinates, and UPGMA clustering analysis assigned each of the three populations to a distinct genetic cluster, corroborating the isolation of the three populations [39].

Considering that the species is hermaphroditic and is pollinated solely through crossbreeding with the help of insects (which have the habit of visiting all the flowers of the same plant [34]), we hypothesize that inbreeding in populations is expected as a consequence of self-fertilization and mating between relatives. These levels of inbreeding and the mating system, coupled with autochoric seed dispersal, anthropogenic disturbances, and the size of the fragments, can determine the spatial genetic structure of *M. nigra* in the semi-deciduous seasonal forest of the Ribeirão Preto region. Additionally, the behavior of pollinators (flies) can easily reach the flowers of many trees, maintaining gene flow within populations.

The present study aimed to study if fragment size, quality, and landscape matrix influence the population structure, inbreeding, and genetic spatial distribution of *M. nigra* across generations in the semi-deciduous seasonal forest remnants of Ribeirão Preto (SP). Additionally, phenological events and germination rates were evaluated in the BSQ-Rib fragment.

## 2. Results

### 2.1. Principal Coordinates Analysis and Analysis of Molecular Variance

**Figure 1:**
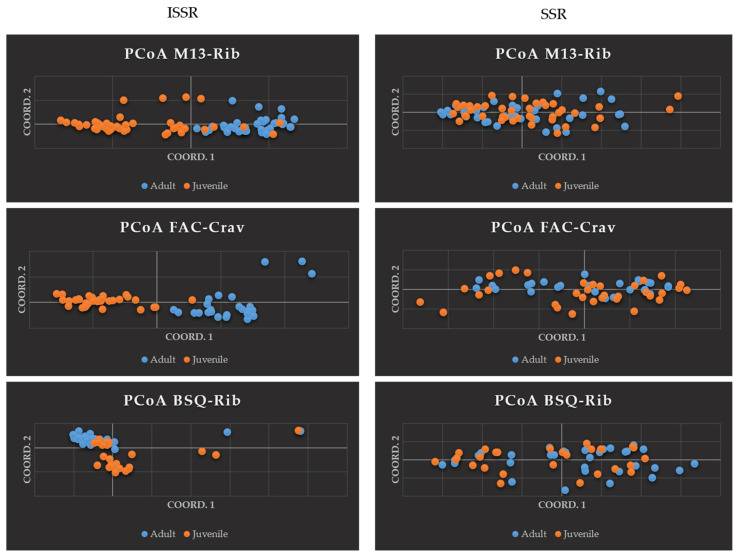
Principal Coordinates Analysis (PCoA) of two generations of *Metrodrea nigra* St. Hill. from three populations in the Ribeirão Preto region (São Paulo, Brazil), performed using ISSR markers (**left**) and SSR markers (**right**).

The Principal Coordinates Analysis (PCoA) showed differences in clustering between generations in the different fragments when studied with ISSR and SSR markers (Figure 1). ISSR markers showed more apparent generational clustering, while SSR markers showed individuals mixed in the plot. According to the Analysis of Molecular Variance (AMOVA), most of the genetic variability is found within the generations; however, greater variability was observed between generations with ISSR markers (M13-Rib 13%, FAC-Crav 19%, and BSQ-Rib 12%) compared to almost no variability with SSR markers (M13-Rib 0%, FAC-Crav 1%, and BSQ-Rib 1%) (Table 1).

### 2.2. Inbreeding and Fst

The analysis of the F-statistic between generations indicated positive and statistically significant values of inbreeding, with higher mean values of Fis (inbreeding within subpopulations) and Fit (overall inbreeding) in BSQ-Rib (0.36, *p* = 0.001) and M13-Rib (0.32, *p* = 0.001), followed by FAC-Crav (0.26, *p* = 0.001) (Table 2). These statistics indicate the degree of consanguinity within a population. High inbreeding values suggest significant matings between close relatives by limited seed dispersal or autogamy, which can lead to inbreeding depression and affect the overall population fitness [2,7]. In contrast, we observed a low F_ST_ (inbreeding due to subdivision or genetic differentiation) and high historical gene flow (Nm), indicating low differentiation between generations in all populations (Table 2).

### 2.3. The Spatial Genetic Structure Between Generations

**Figure 2:**
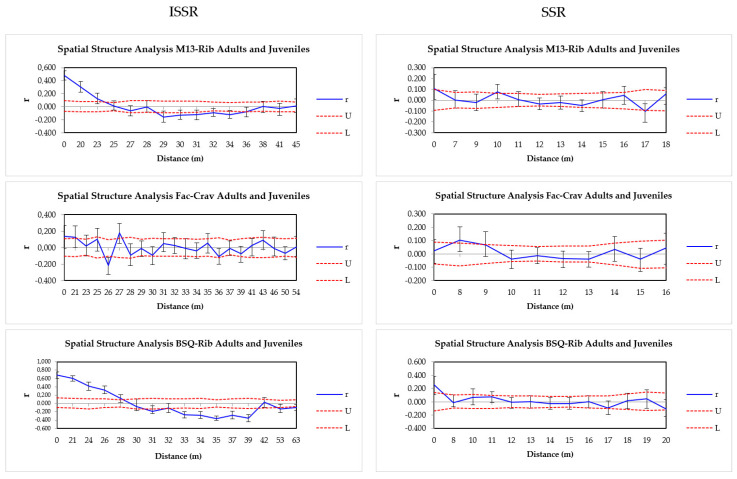
Spatial Autocorrelation Correlograms for M13-Rib, FAC-Crav, and BSQ-Rib from ISSR (**left**) and SSR (**right**) marker data considering adult and juvenile generations. The upper (U) and lower (L) red dashed lines represent the 95% confidence interval for *r* = 0, and the vertical bars indicate the 95% confidence interval for each *r*-value. Blue lines outside the confidence interval denote *r*-values significantly different from 0, indicating genetic structuring.

Analyzing the data from both markers, SGS was detected in the first distance classes (21–28 m [ISSR], 0–8 m [SSR]), considering the two generations as one population (Table 3, Figure 2). The highest r values were recorded in adults and juveniles of the BSQ-Rib population, indicating higher SGS in these samples (26 m juveniles, 24 m adults [ISSR]; 7 m juveniles, 9 m adults [SSR]) (Table 3, Supplementary Figures S1 and S2). In M13-Rib, SGS was detected in the first distance class (19 m in juveniles and 24 m in adults [ISSR]; 0 m in juveniles and 10 m in adults [SSR]). In FAC-Crav, SGS was not observed in adults with both molecular markers; however, in the juvenile generation, SGS was identified punctually at 27 m (ISSR) and 8 m (SSR) (Table 3, Supplementary Figures S1 and S2). When analyzing FAC-Crav generations with ISSR, SGS was found at 21 m (Table 3, Figure 2).

In general, we observed significant spatial genetic structuring (SGS) with both ISSR and SSR markers, particularly in the first distance classes across all populations, with ISSR markers showing higher SGS. The highest r values were seen in the BSQ-Rib population for adults and juveniles, indicating higher SGS in these samples (Supplementary Figures S1 and S2). Additionally, negative SGS was observed punctually in all populations between 26 and 39 m (ISSR) and between 9 and 14 m in BSQ-Rib juveniles and M13-Rib adults (SSR) (Supplementary Figures S1 and S2), suggesting that individuals at these distances are genetically less similar. The differences in the analyses with both markers show that the ISSR markers captured variations in a greater diversity of *M. nigra* genomic regions compared to those covered by the SSRs used.

### 2.4. Phenology of Metrodorea nigra in BSQ-Rib

**Figure 3:**
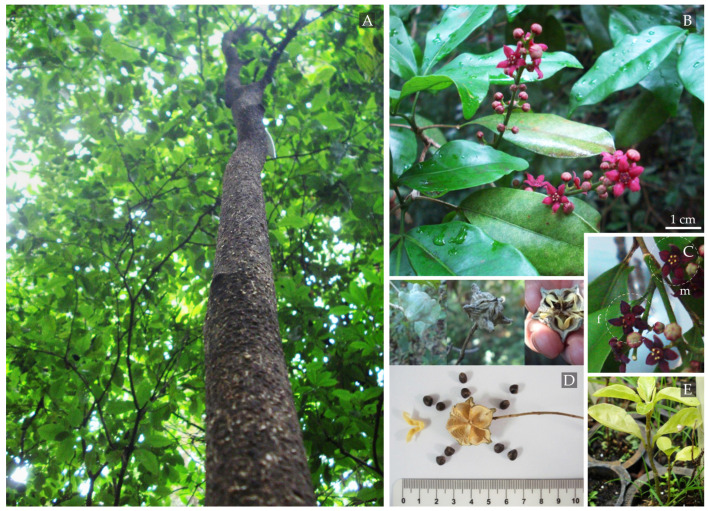
*Metrodorea nigra* species in the BSQ-Rib Forest fragment in Ribeirão Preto, SP, Brazil. Leaves, trunk, and pods (**A**). New open flowers (**B**) and in male [m] and female [f] phases (**C**), fruits and seeds (**D**). (**E**) *M. nigra* germination in the USP/RP nursery.

Initially, dry aged fruits were observed in adult trees and two juveniles (Juv9 and Juv10), indicating previous fruiting phases in this fragment. By the end of December, we observed the conclusion of the new leaf sprouting phase and the onset of the flowering phase, with the development of flower buds in both adult and juvenile individuals (Table 4, Figure 3). In January, during the rainy and warm seasons, the marked adult trees, the two juveniles, and the general adult trees in the BSQ-Rib fragment exhibited abundant flowering and intense insect visitation. However, the slight smell of rotten meat in *M. nigra* flowers, previously reported by Pombal and Morellato [34] and by our group in the FAC-Crav fragment [38], was not detected.

The newly opened *M. nigra* flowers were violet (Figure 3B,C). As they aged, we observed the male and female phases, and the color transitioned to darker shades, turning burgundy, brown, and black until the withering and fall of the flowers occurred over approximately 60 days (Figure 3). According to climate data from the Integrated Center for Agrometric Information (CIIAGRO) [40], the peak flowering period of *M. nigra* occurred between January and February at an average temperature of 25 °C, with 66% average relative humidity and 194 mm of rainfall (Table 4). Other flowering periods of *M. nigra* were reported from October to November (1991–1993) in Campinas, SP, Brazil [34], and from July to January (1999–2000) with a peak in August–September in Maringá, PR, Brazil [35].

No apparent changes were noticed in the trees except continuous leaf loss in the following months. In August, fruit development was observed in adult and mature individuals, with two juvenile individuals being highly reproductive. Some adult individuals, not necessarily the ones being monitored, also showed fruit development in August. Trees located on the edge and near internal trails of the fragment produced the most significant number of fruits, likely due to easier access for pollinators. However, in general, few fruits were detected, in contrast to the profuse blossoming during the flowering period, as also reported by Pombal and Morelatto [34]. The ripening phase of the fruits was observed during a particularly dry period of the year, in August (temperature 22 °C, RH 49, and precipitation of 3 mm), and extended to September (temperature 24 °C, RH 58, and precipitation of 93 mm) (Table 4). This period, characterized by lower rainfall, facilitated the drying of the fruits and the subsequent release of seeds, as also reported by Pombal and Morellato from 1991 to 1993 [34].

In the flowers of 12 adult trees that were covered from the beginning of flowering until the fruit development phase, no fruit production was observed, highlighting the importance of insect visits for pollination. Leaf fall was frequent throughout the year, with higher intensity between March and April (Table 4). This leaf litter brings great benefits to all trees in the forest due to its nutrient and organic compound content, making *M. nigra* a key species in forest nutrient dynamics [26].

#### Seed Collection and Germination

**Figure 4:**
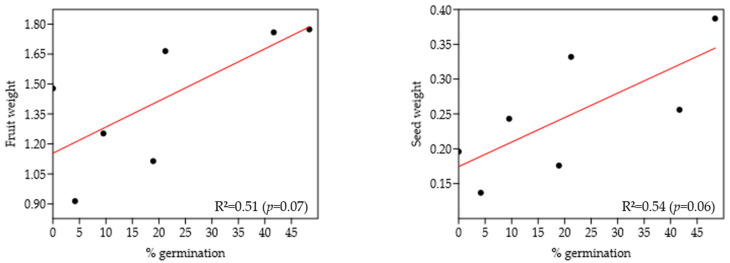
Regression analysis of the variable fruit weight vs. % of germination (**left**) and the variable seed weight vs. % of germination (**right**). R^2^ corresponds to the correlation coefficient.

A total of 215 seeds were obtained from the 36 fruits collected. The average weight of fruits and seeds was 1.42 g and 0.25 g, respectively (Figure 3D, Table 5). After 40 days of planting, the first germination of some seeds was observed. After 60 days, 20% of the seeds germinated (47 seeds), while 79.5% did not (169 seeds). None of the 33 seeds from individual Mtn11 germinated. Mtn16, with the lowest weight of fruits and seeds (0.914 and 0.137 g, respectively), had the lowest germination (4.17%), whereas individuals Juv9 and Mtn13 with the heaviest fruits (Juv9: 1.77 g; Mtn13: 1.76 g) showed the highest germination percentages (48.38% and 41.66%, respectively). Regression analyses between fruit weight and seed weight vs. seed germination percentage showed a moderate correlation, indicating that the heaviest fruits produced larger seeds with higher germination percentages. However, this inference should be taken cautiously, given the lack of strong statistical significance (*p* > 0.05) (Figure 4).

## 3. Discussion

Previous studies by our group in the same populations from the three fragments showed high levels of genetic diversity (*He*_BSQ-Rib_ = 0.27, *He*_M13-Rib_ = 0.26, and *He*_FAC-Crav_ = 0.24 with ISSR markers; *He*_BSQ-Rib_ = 0.765, *He*_M13-Rib_ = 0.704, and *He*_FAC-Crav_ = 0.688 with SSR markers) and low separation among populations (Fst = 0.062). The FAC-Crav and BSQ-Rib populations showed more considerable genetic similarities to each other than compared to the M13-Rib population [39]. Extending these findings, in this manuscript, we present a fine-scale genetic analysis among generations in each of these populations to study genetic structuring, inbreeding, and spatial genetic structure. Additionally, in the smallest fragment (BSQ-Rib, 3 ha), we studied the phenophases, seed production, and germination of *Metrodorea nigra* species.

The present analysis, particularly with ISSR markers, revealed that the two generations of the three populations tended to form distinct groups near each other. Specifically, 12% (FAC-Crav), 13% (M13-Rib), and 19% (BSQ-Rib) of the variance were attributed to generational separation. However, most variance is within generations, as indicated by the very low Fst values and high historical gene flow (Nm), suggesting no significant separation between generations, which may be a consequence of outcrossing that promotes pollen movement between individuals, reducing differentiation within species [2].

**Figure 5:**
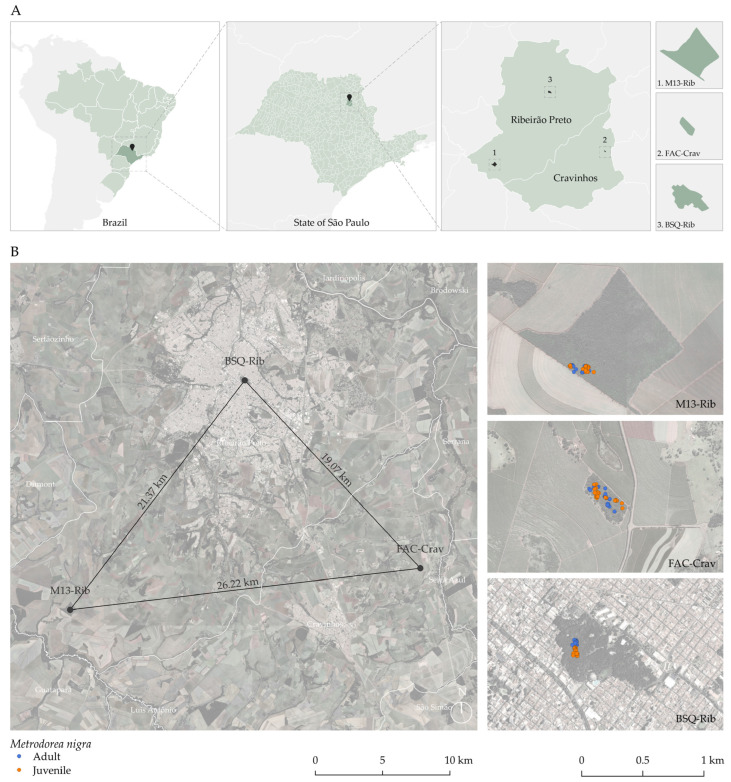
Localization (**A**) and distribution (**B**) of the three studied *Metrodorea nigra* St. Hill. populations in forest fragments of Ribeirão Preto, São Paulo, Brazil. The graph map on the left shows distances between forest fragments. On the right of B, three zoomed-in views display adult (blue) and juvenile (orange) individuals in each fragment. All maps were created in QGIS 3 using field-collected data and Google Earth satellite imagery.

Confirming our hypotheses, it is noteworthy that the levels of inbreeding (Fis and Fit) among the generations of all populations were positive and statistically significant, which can be attributed to fragmentation, autochorous seed dispersal, and pollinator behavior. On the other hand, at a micro-scale level, our *M. nigra* SGS analysis shows that the size of the forest fragment is not the only determining factor. Although the 8 ha FAC-Crav fragment is smaller than the M13-Rib (84 ha), it showed low spatial genetic structure among the studied generations, making it an interesting site for seed collection for reforestation. Additionally, inbreeding, anthropogenic disturbances such as past selective logging, and the existence of forest trails in the urban Municipal Forest where the *M. nigra* population occurs [20] may cause the highest SGS observed in the BSQ-Rib population. Our phenological observations showed that, despite profuse flower production in the BSQ-Rib population, the low fruit and seed number and low germination rates may be consequences of SGS in response to inbreeding, limited pollinators in the forest interior, mating system, autochorous seed dispersal, and self-incompatibility mechanisms. The matrix surrounding the fragments also has considerable consequences for the SGS of the populations [41]. In the studied fragments, the M13-Rib and FAC-Crav populations are surrounded by sugarcane plantations, whereas the BSQ-Rib population is within a high human density matrix (see Figure 5). Overall, the spatial genetic structure observed in the three fragments was more pronounced over short distances.

*Metrodorea nigra* has been described as an allogamous and self-incompatible species in floral biology studies [34]. We also did not observe fruiting from the bagged flowers in our phenological observations, showing the necessity of pollinators for fruiting. According to Vekemans and Hardy [3], the SGS of forest species is significantly related to their mating system and population density, with allogamous species and high-density populations exhibiting less genetic structure. However, anthropogenic disturbances such as canopy opening and selective logging (M13-Rib) can fragment populations, further reducing gene flow and increasing inbreeding and SGS [26,29]. The inbreeding and spatial genetic structure resulting from mating between relatives, self-fertilization, limited seed dispersal, and disturbances are consistent with expectations for species with entomophilous pollination, pollinator behavior with visits to the flowers of the same plant, and autochorous dispersal, as is the case of *M. nigra*.

In summary, the data obtained in this research highlight the effects of fragmentation and isolation on the SGS of *Metrodorea nigra* in three forest fragments of the Ribeirão Preto Region (SP, Brazil). The findings show that anthropogenic disturbances, in addition to fragmentation and the matrix in which the fragments are immersed, are important factors that shape the resilience of these populations by directly affecting pollinator survival and behavior [26,29]. In turn, these factors—together with natural seed dispersal, mating among relatives, and pollinator geitonogamy—intensify inbreeding and SGS in *M. nigra* populations, potentially impacting their reproductive success and long-term survival in the face of climate change.

## 4. Materials and Methods

### 4.1. Selection of Remnants of the Semi-Deciduous Seasonal Forest for Spatial Analysis

We selected the M13-Rib and FAC-Crav fragments on private properties, as well as the BSQ-Rib fragment in the Municipal Park of Morro de São Bento, which has a disturbance history dating back to the 19th century, in the Region of Ribeirão Preto, SP, Brazil (Table 6, Figure 5). These fragments, surrounded by different matrices and equidistantly located, vary in size (Table 6, Figure 5).

The fragments host populations of *M. nigra*, including clusters of juvenile and adult individuals occurring very close (between 1 and 7 m), along with a few isolated ones. The distances between the trees ranged from 0 to 40 m (Supplementary Figure S3). We observed offspring in all fragments, mainly near the edges and/or trails where they received higher light. Although not counted, offspring were most evident in number at BSQ-Rib, followed by FAC-Crav.

We registered the GPS coordinates (Garmin eTrex Vista Cx, Garmin International, Olathe, KS, USA) of all individuals with samples collected in the three fragments (M13-Rib: 37 adults, 43 juveniles; FAC-Crav: 27 adults, 33 juveniles; BSQ-Rib: 32 adults, 28 juveniles), aiming to study the spatial structure of these populations. The individuals sampled from the three selected fragments were classified as adults or juveniles according to Perimeter at Breast Height (PBH), with juveniles defined as individuals with PBH < 11 cm and adults with PBH ≥ 11 cm. Prof. Dr. Milton Groppo Jr. identified the species and collected an exsiccate (SPFR 12706) at the FAC-Crav fragment (Groppo collector # 1971) and deposited it in the Herbarium of the Biology department (USP/FFCLRP). We previously studied the genetic diversity of the 200 adult and juvenile individuals sampled from the three selected fragments [39].

### 4.2. Plant Material, DNA Extraction, and Molecular Markers

We extracted DNA from leaf samples collected from 200 individuals from the three populations, following the protocol described by Alzate-Marin et al. (2009) [42]. We used 8 ISSR primers (UBC 1, 2, 820, 834, 851, 858, 860, 886) selected from the University of British Columbia’s official list (UBC), which produced amplification products in other plant species [43] and showed polymorphic bands in *M. nigra* (Supplementary Table S1). Additionally, we selected 7 SSR markers (Mtn 1, 3, 13, 16, 19, 87, 95) from the 15 SSR markers developed for the species by our group [38,44] due to their polymorphism and clear amplification quality (Supplementary Table S2). The ISSR/SSR amplification conditions are described in Moraes Filho et al. (2015) [39].

### 4.3. Phenology and Seed Germination of M. nigra in the BSQ-Rib Fragment

We randomly marked ten juveniles (PBHmean = 14) and ten adult individuals (PBHmean = 30) both in the interior and at the edge of the fragment, within an approximate radius of 60 m. The distances between the trees ranged from 0 to 20 m. Over 12 months (November 2014 to November 2015), we conducted weekly phenological observations between 10 and 12 a.m. to record data on leaf sprouting and falling, flowering, and fruiting development. To confirm these observations, the study area was systematically surveyed, evaluating the occurrence of the phenophase in the marked individuals and the surrounding trees, aiming for a broader scope of the recorded characteristics.

To verify seed formation without insect visitation, we bagged flowers from 12 trees until the end of the period in March 2015.

At the end of fruit maturation, we bagged the fruits to prevent seed loss and complete their ripening, and after a week, we collected, identified, and preserved them in paper bags to avoid moisture. We exposed the fruits to the sun for six days, and in the end, the seeds were naturally released. We sowed the seeds in the seedling nursery at the University of São Paulo, Ribeirão Preto Campus. We planted each seed in a tube containing a mix of pine, coconut fiber, and vermiculite, identified by family.

### 4.4. Statistical Analysis

#### 4.4.1. Inbreeding and Genetic Structure

We used GenAlex 6.5 software version 6.5b2 [45] to estimate the inbreeding coefficient (Fis and Fit) and genetic differentiation (Fst) between generations (adult and juvenile) of each population using SSR amplifications. The statistical significance for these analyses was determined through 999 bootstraps. Additionally, we used this software to identify genetic structure patterns with principal coordinate analysis and molecular variance analysis (AMOVA) to quantify the distribution of genetic diversity between generations using both ISSR and SSR molecular markers.

Regression analyses were conducted between fruit weight and seed weight versus seed germination percentage (dependent variable) using PAST software version 4.03 [46], aiming to identify the relationship between these variables.

#### 4.4.2. Spatial Genetic Structure

Due to the limited mobility of plants, their genetic structure inherently implies a spatial structure or the physical distribution of individuals [2]. Many ecological and evolutionary factors influencing genetic variation are mediated by space, so joint analysis of genetic and spatial information can enhance our understanding by investigating the relationship between geographic and genetic distances. These concepts are crucial in molecular ecology for detecting, quantifying, and testing the spatial structure of genetic variation. Additionally, landscape genetics aims to elucidate how genetic variation is affected by landscape and environmental variables [47].

We performed the spatial autocorrelation analysis (SGS) using genetic distance matrices obtained through ISSR and SSR markers, compared to matrixes of geographic distance between individuals calculated by their GPS coordinates using the GenAlex 6.5 software [45]. SGS executed a multivariate analysis combining loci rather than individual loci [48]. An autocorrelation coefficient (r) was generated between genetic and geographic distances within user-defined distance classes. The coefficient r measures the genetic similarity between pairs of sampled individuals whose geographic separation falls within a specific distance class. This correlation coefficient ranges from −1 to 1, where 0 means no autocorrelation. Values above 0 indicate positive autocorrelations, and values below 0 indicate negative autocorrelations. Statistical significance tests for r in each distance class were performed with 1000 random permutations and bootstrapping, as described by Peakall et al. [49].

## 5. Conclusions

The fragment size did not influence the levels of inbreeding in the *M. nigra* populations, which is probably intensified by fragmentation, the species’ limited seed dispersal system (autochory), and pollinator behavior favoring geitonogamy, leading to the formation of clusters. Conversely, pollen gene flow likely acts as a population homogenizer, facilitating the exchange of alleles between individuals and maintaining levels of diversity between generations, indicating that current pressures have not altered the genetic composition of the analyzed generations. Therefore, pollinator behavior and seed dispersal are critical in shaping genetic spatial structure and reproductive success in *M. nigra*.

SGS (Spatial Genetic Structure) in all populations results from inbreeding, enhanced by anthropogenic disturbances, a history of selective logging, trails, and the surrounding urban matrix, as observed in BSQ-Rib. However, fragment size did not affect SGS since the FAC-Crav fragment (8 ha) shows low SGS despite being smaller than the M13-Rib fragment (84 ha). Larger fragments do not necessarily harbor more genetically intact populations. The conservation status and disturbance history are relevant factors. Thus, regardless of the fragment size, the spatial distribution of seed tree genotypes following logging can affect the genetic constitution of regeneration.

The FAC-Crav fragment is a better source for seed collection. This population does not exhibit SGS, is preserved, and presents a high number of species, especially tall tree species such as *Aspidosperma polyneuron* [50] and *Cariniana estrellensis* [51], under whose shade *M. nigra* occurs. Previous genetic diversity studies show that the FAC-Crav population possesses substantial diversity, though lower than that of BSQ. Additionally, distance genetic studies show that FAC-Crav and BSQ-Rib are part of one major group [39]. Therefore, seed collections for reforestation purposes should be conducted in both populations. These results highlight the relevance of this information for the measurement and management of the genetic composition of forest populations.

## Figures and Tables

**Table 1:** Analysis of Molecular Variance (AMOVA).

AMOVA	M13-Rib ISSR	M13-Rib SSR	FAC-Crav ISSR	FAC-Crav SSR	BSQ-Rib ISSR	BSQ-Rib SSR
Among generations	13%	0%	19%	1%	12%	1%
Within generations	87%	100%	81%	99%	88%	99%
Total	100%	100%	100%	100%	100%	100%
*p*-values	0.001 *	0.712	0.001 *	0.210	0.001 *	0.131

* *p*: Significance to 95%.

**Table 2:** F-Statistics for two generations from three *M. nigra* St. Hill. populations.

	M13-Rib		FAC-Crav		BSQ-Rib SSR	
	Value	*p*-Values	Value	*p*-Values	Value	*p*-Values
Fst	−0.003	0.806	0.004	0.155	0.007	0.074
Fis	0.317	0.001 *	0.256	0.001 *	0.356	0.001 *
Fit	0.315	0.001 *	0.259	0.001 *	0.360	0.001 *
Nm	-	-	59	-	35	-

* *p*: Significance to 95%.

**Table 3:** Characterization of the spatial genetic structure of the three *M. nigra* fragments in two generations. N: Number of individuals, Int: Distance from the first intersection to the *x*-axis, *r*: Spatial autocorrelation coefficient in the first distance class, ^&^ SGS identified punctually, * p: Significance to 95%.

Marcador	População	N	Int	*r*	*p*-Values
ISSR all generations	M13-Rib	80	23	0.13	0.002 *
	FAC-Crav	60	21	0.13	0.016 *
	BSQ-Rib	60	28	0.12	0.005 *
SSR all generations	M13-Rib	80	0	0.11	0.022 *
	FAC-Crav	60	8 ^&^	0.10	0.011 *
	BSQ-Rib	60	0	0.23	0.001 *
ISSR Adult/juvenile	M13-Rib (adult)	37	24	0.24	0.006 *
	M13-Rib (Juvenile)	43	19	0.13	0.020 *
	FAC-Crav (adult)	27	-	-	-
	FAC-Crav (Juvenile)	33	27 ^&^	0.28	0.005 *
	BSQ-Rib (adult)	32	24	0.34	0.003 *
	BSQ-Rib (Juvenile)	28	26	0.21	0.026 *
SSR Adult/juvenile	M13-Rib (adult)	37	10	0.16	0.024 *
	M13-Rib (Juvenile)	43	0	0.34	0.001 *
	FAC-Crav (adult)	27	-	-	-
	FAC-Crav (Juvenile)	33	8 ^&^	0.15	0.031 *
	BSQ-Rib (adult)	32	9	0.18	0.012 *
	BSQ-Rib (Juvenile)	28	7	0.42	0.001 *

**Table 4:** Sprouting, flowering, fruiting, and leaf fall observed in *Metrodorea nigra* in adult (A) and juvenile (J) individuals in the BSQ-Rib Forest fragment in Ribeirão Preto (RP), São Paulo (SP), Brazil.

	2014		2015										
	Nov	Dec	Jan	Feb	Mar	Apr	May	Jun	Jul	Aug	Sep	Oct	Nov
Leaf sprouting	A-J	A-J											
Floral buds		A-J *											
Flower opening			A-J *	A	A								
Leaf falling	L	L	L	L	H	H	L	L	L	L	L	L	L
Beginning of fruiting										A-J *	A		
Fruit ripening											A-J *	A	
AT (°C) ^1^	25	25	26	25	24	23	20	20	20	22	24	27	25
ARH (%) ^2^	64	68	62	69	75	69	70	65	65	49	58	54	70
AP (mm) ^3^	111	240	148	239	178	29	94	26	14	3	93	53	210

* Only two Juvenile individuals 9 and 10, L = constant and lower leaf falling, H = high leaf falling, ^1^ Average Temperature, ^2^ Average Relative humidity, ^3^ Average Precipitation, (http://www.ciiagro.org.br/mensal/cmensal, (accessed on 14 February 2025) 2016 [40]).

**Table 5:** Individuals, location, the number of mature fruits (FN), and quantity of seeds collected (SN) from the species *M. nigra*, in the Municipal Park of Morro de São Bento RP/SP (BSQ-Rib). WDF = Weight of dried fruit, WDS = Weight of dried seeds, NGS = number of germinated seeds, NUS = number of ungerminated seeds. SD = standard deviation. Ψ GPS accuracy in meters (m).

Indivídual	GPS	FN	SN	WDF (g)	WDS (g)	NGS	NUS	Germination (%)
JUV09	S 21°10′20.0″ W 047°48′05.6″ (^Ψ^ 7 m)	5	31	1.77	0.39	15	16	48.38
JUV10	S 21°10′20.1″ W 047°48′05.7″ (^Ψ^ 5 m)	3	21	1.25	0.24	2	19	9.52
MTN11	S 21°10′18.3″ W 047°48′07.7″ (^Ψ^ 5 m)	5	33	1.48	0.20	0	33	0.00
MTN12	S 21°10′18.8″ W 047°48′08.2″ (^Ψ^ 7 m)	5	33	1.67	0.33	7	27	21.21
MTN13	S 21°10′19.8″ W 047°48′05.6″ (^Ψ^ 8 m)	6	36	1.76	0.26	15	21	41.66
MTN14	S 21°10′20.2″ W 047°48′05.6″ (^Ψ^ 7 m)	6	37	1.11	0.18	7	30	18.92
MTN16	S 21°10′21.0″ W 047°48′05.6″ (^Ψ^ 6 m)	6	24	0.91	0.14	1	23	04.17
	Total	36	215	9.95	1.74	47	169	144
	Average	5.14	30.71	1.42	0.25	6.71	24.14	20.55
	SE	0.40	2.28	0.13	0.03	2.38	2.31	6.96

**Table 6:** Characteristics of the three forest fragments selected to study the species *Metrodorea Nigra* St. Hill. in the Region of Ribeirão Preto, SP, Brazil ^&^.

Fragment	Area (ha)	Altitude/GPS	Characteristics	Access	Matrix
M13-Rib	84	601 m (21°19′45.90″ S; 47°55′30.38″ W)	Located at “Bomba” Farm (Ribeirão Preto, SP), M13 is in better condition than other nearby large fragments, hosting a population of *M. nigra* in a denser region of the forest, especially in the shade of larger trees. Part of the forest is regenerating after a process of fire and selective logging. Large portions of this fragment exhibit edge effects and the presence of lianas. We sampled all adult individuals in this population.	Open/private property	Extensive sugarcane plantations
FAC-Crav	8	610 m (21°17′54.22″ S; 47°40′28.10″ W)	This forest fragment at “Águas Claras” farm (Cravinhos—SP) appears to be in well-preserved conservation condition due to being fenced and having less contact with human populations. We observed a few clearings, and we did not find signs of burning. The collected individuals occur throughout the fragment, in the shade of large trees, especially ancient *Aspidosperma polyneuron* and *Cariniana estrellensis* trees.	Closed /private property	Extensive sugarcane plantations
BSQ-Rib	3	570 m (21°10′26.37″ S; 47°48′1.49″ W)	The semi-deciduous forest fragment, where the *M. nigra* population is found, is located along a trail and on the hillside within the area of the Municipal Park of Morro de São Bento. The park covers a total of 18 hectares and is situated in the middle of Ribeirão Preto city, SP. The fragment exhibits several clearings and is visited by people for educational activities, having a history of selective logging up until the 1960s. The collected individuals occur throughout the fragment, especially in the shade of tall trees.	Closed /public	Intensely urbanized

^&^ Modified from Moraes Filho et al., 2015 [39].

## Data Availability

Data are unavailable due to privacy.

## Supplementary Materials

The following supporting information can be downloaded at https://www.mdpi.com/article/10.3390/plants14050702/s1: Figure S1. Spatial Autocorrelation Correlograms for M13-Rib, FAC-Crav, and BSQ-Rib from ISSR marker data considering adult and juvenile generations. The upper (U) and lower (L) red dashed lines represent the 95% confidence interval for *r* = 0, and the vertical bars indicate the 95% confidence interval for each *r*-value. Blue lines outside the confidence interval denote *r*-values significantly different from 0, indicating genetic structuring. Figure S2. Spatial Autocorrelation Correlograms for M13-Rib, FAC-Crav, and BSQ-Rib from SSR marker data considering adult and juvenile generations. The upper (U) and lower (L) red dashed lines represent the 95% confidence interval for *r* = 0, and the vertical bars indicate the 95% confidence interval for each *r*-value. Blue lines outside the confidence interval denote *r*-values significantly different from 0, indicating genetic structuring. Figure S3. Frequency and distance to the nearest neighbor from the first individual for the M13-Rib, FAC-Crav, and BSQ-Rib populations. Table S1. Polymorphism of ISSR primers used (Moraes Filho et al., 2015). PL: Polymorphic Loci; ML: Monomorphic Loci; ĤE = Nei genetic diversity. AT= Annealing Temperature. Table S2. Characteristics of the *M. nigra* microsatellite loci. Na = number of alleles; Ne = effective number of alleles; Ho = observed heterozygosity; He = expected heterozygosity; F = fixation index. AT= Annealing Temperature (Moraes Filho et al., 2015).

## Abbreviations

The following abbreviations are used in this manuscript:

AMOVA	Analysis of Molecular Variance
ISSR	Inter Simple Sequence Repeat
SSR	Simple sequence repeats
PCoA	Principal Coordinates Analysis

## References

[1] Legendre P., Fortin M.J. (1989). Spatial Pattern and Ecological Analysis. Vegetatio.

[2] Loveless M.D., Hamrick J.L. (1984). Ecological Determinants of Genetic Structure in Plant Populations. Annu. Rev. Ecol. Syst..

[3] Vekemans X., Hardy O.J. (2004). New Insights from Fine-scale Spatial Genetic Structure Analyses in Plant Populations. Mol. Ecol..

[4] Nazareno A.G., Jump A.S. (2012). Species–Genetic Diversity Correlations in Habitat Fragmentation Can Be Biased by Small Sample Sizes. Mol. Ecol..

[5] Epperson B.K. (1992). Spatial Structure of Genetic Variation within Populations of Forest Trees. Proceedings of the Population Genetics of Forest Trees: Proceedings of the International Symposium on Population Genetics of Forest Trees.

[6] Shapcott A. (1995). The Spatial Genetic Structure in Natural Populations of the Australian Temperate Rainforest Tree *Atherosperma moschatum* (Labill.) (Monimiaceae). Heredity.

[7] Young A., Boyle T., Brown T. (1996). The Population Genetic Consequences of Habitat Fragmentation for Plants. Trends Ecol. Evol..

[8] Hardy O.J., Maggia L., Bandou E., Breyne P., Caron H., Chevallier M., Doligez A., Dutech C., Kremer A., Latouche-Hallé C. (2006). Fine-scale Genetic Structure and Gene Dispersal Inferences in 10 Neotropical Tree Species. Mol. Ecol..

[9] Robledo-Arnuncio J.J., Klein E.K., Muller-Landau H.C., Santamaría L. (2014). Space, Time and Complexity in Plant Dispersal Ecology. Mov. Ecol..

[10] Fournier L.A. (1976). El Dendrofenograma, Una Representación Gráfica Del Comportamiento Fenológico de Los Árboles. Turrialba.

[11] Morellato L.P.C. (1991). Estudo Da Fenologia de Arvores, Arbustose Lianas de Uma Floresta Semidecidua Do Sudeste Do Brasil. Ph.D. Thesis.

[12] Morellato L.P.C. (1995). Ecologia e Preservação de Uma Floresta Tropical Urbana: Reserva de Santa Genebra.

[13] Morellato L.P.C., Altomare M., Gressler E., Schwartz M.D. (2024). A Review of Reproductive Plant Phenology in South and Central America: New Perspectives BT—Phenology: An Integrative Environmental Science.

[14] Ghazoul J. (2005). Pollen and Seed Dispersal among Dispersed Plants. Biol. Rev..

[15] Willi Y., Van Buskirk J., Schmid B., Fischer M. (2007). Genetic Isolation of Fragmented Populations Is Exacerbated by Drift and Selection. J. Evol. Biol..

[16] Neaves L., Eales J., Whitlock R., Hollingsworth P.M., Burke T., Pullin A. (2015). The fitness consequences of inbreeding in natural populations and their implications for species conservation—A systematic map. Environ. Evid..

[17] de Jong T.J., Waser N.M., Klinkhamer P.G. (1993). Geitonogamy: The neglected side of selfing. Trends Ecol. Evol..

[18] Morellato L.P.C., Haddad C.F.B. (2000). Introduction: The Brazilian Atlantic Forest 1. Biotropica.

[19] Ribeiro M.C., Metzger J.P., Martensen A.C., Ponzoni F.J., Hirota M.M. (2009). The Brazilian Atlantic Forest: How Much Is Left, and How Is the Remaining Forest Distributed? Implications for Conservation. Biol. Conserv..

[20] Kotchetkoff-Henriques O. (2003). Characterization of the Natural Vegetation in Ribeirão Preto, SP: Bases for Conservation. DSc thesis.

[21] Carvalho S., Varanda E.M. (2008). Biodiversidade e Pioneirismo Na Floresta Da USP Ribeirão Preto. Fecunda. https://biofecunda.wordpress.com/2008/10/23/biodiversidade-e-pioneirismo-na-floresta-da-usp-ribeirao-preto/.

[22] Eckert C.G., Kalisz S., Geber M.A., Sargent R., Elle E., Cheptou P.-O., Goodwillie C., Johnston M.O., Kelly J.K., Moeller D.A. (2010). Plant Mating Systems in a Changing World. Trends Ecol. Evol..

[23] Durigan G., Franco G.A.D.C., Saito M., Baitello J.B. (2000). Estrutura e Diversidade Do Componente Arbóreo Da Floresta Na Estação Ecológica Dos Caetetus, Gália, SP. Braz. J. Bot..

[24] Metzger J.P. (2000). Tree Functional Group Richness and Landscape Structure in a Brazilian Tropical Fragmented Landscape. Ecol. Appl..

[25] Bertoncini A.P. (2003). Estrutura e Dinamica de Uma Area Perturbada Na Terra Indigena Arariba (Avai, SP): Implicações Para o Manejo e a Restauração Florestal. https://ssbr.oa.mg/work/10.47749/t/unicamp.2003.290969.

[26] Villela D.M., Nascimento M.T., de Aragao L.E.O.C., Da Gama D.M. (2006). Effect of Selective Logging on Forest Structure and Nutrient Cycling in a Seasonally Dry Brazilian Atlantic Forest. J. Biogeogr..

[27] Cassola H. (2008). Aspectos Da Estrutura Fitossociológica e Silvigenética Em Fragmentos de Floresta Estacional Semidecídua Com Diferentes Histórias de Perturbação Em Botucatu. Ph.D. Thesis.

[28] Marcondelli A.C.B. (2010). Estrutura de Uma Comunidade Arbórea de Floresta Estacional Semidecídua Não Pertubada No Noroeste Paulista Em Relação à Outra Comunidade Com Indicadores de Perturbação. Master’s Thesis.

[29] Schwarcz K.D., Pataca C.L., Abreu A.G., Bariani J.M., Macrini C.M.T., Solferini V.N. (2010). Genetic Diversity in Atlantic Forest Trees: Fragmentation Effects on *Astronium Graveolens* (Anacardiaceae) and *Metrodorea Nigra* (Rutaceae), Species with Distinct Seed Dispersal Strategies. Bot. J. Linn. Soc..

[30] Dias P., Udulutsch R.G., Pirani J.R. (2015). Molecular Phylogeny and Biogeography of the South American Genus Metrodorea (Rutaceae). Turk. J. Bot..

[31] Tanaka G.K., Groppo M. (2009). Estrutura e Florística Do Estrado Arbóreo de Um Fragmento de Floresta Estacional Semidecidual: Estação Ecológica de Ribeirão Preto. Ph.D. Thesis.

[32] Capretz R.L., Batista J.L.F., Sotomayor J.F.M., da Cunha C.R., Nicoletti M.F., Rodrigues R.R. (2012). Padrão Espacial de Quatro Formações Florestais Do Estado de São Paulo, Através Da Função K de Ripley. Ciência Florest..

[33] Lorenzi H. (2002). Árvores Brasileiras: Manual de Identificação e Cultivo de Espécies Arbóreas Nativas Do Brasil.

[34] Pombal E.C.P., Patrícia L., Morellato C. (2000). Differentiation of Floral Color and Odor in Two Fly Pollinated Species of Metrodorea (Rutaceae) from Brazil. Plant Syst. Evol..

[35] de Souza L.A., Moscheta I.S., Mourão K.S.M., Rosa S.M. (2004). da Morphology and Anatomy of the Flower and Anthesis of Metrodorea Nigra St. Hill.(Rutaceae). Braz. Arch. Biol. Technol..

[36] Barbosa, Luiz M., Regina T.S., Fernando C.D.L., Paulo R.T.O., Karina C.B., Tiago C.B. (2017). Lista de espécies indicadas para restauração ecológica para diversas regiões do Estado de São Paulo/Luiz Mauro Barbosa.

[37] de Souza L.A., da Rosa S.M., Moscheta I.S. (2008). Anatomy of the Developing Fruit of Metrodorea Nigra A. St.-Hil.(Rutaceae). Braz. Arch. Biol. Technol..

[38] Guidugli M.C., Ferreira-Ramos R., de Sousa A.C.B., Cidade F.W., Marconi T.G., Mestriner M.A., Groppo M., Alzate-Marin A.L. (2012). Genetic Diversity of *Metrodorea Nigra* (Rutaceae) from a Small Forest Remnant in Brazil Assessed with Microsatellite Markers. Genet. Mol. Res..

[39] Moraes Filho R.M., Bonifácio-Anacleto F., Alzate-Marin A.L. (2015). Fragmentation Effects and Genetic Diversity of the Key Semidecidual Forest Species Metrodorea Nigra in Southwestern Brazil. Genet. Mol. Res..

[40] (2025). CIIAGRO—Portal Agrometeorológico e Hidrológico do Estado de São Paulo. http://www.ciiagro.org.br/mensal/cmensal.

[41] Guiller A., Decocq G., Kichey T., Poli P., Vandepitte K., Dubois F., Honnay O., Closset-Kopp D. (2023). Spatial Genetic Structure of Two Forest Plant Metapopulations in Dynamic Agricultural Landscapes. Landsc. Urban Plan..

[42] Alzate-Marin A.L., Guidugli M.C., Soriani H.H., Martinez C.A., Mestriner M.A. (2009). An Efficient and Rapid DNA Minipreparation Procedure Suitable for PCR/SSR and RAPD Analyses in Tropical Forest Tree Species. Braz. Arch. Biol. Technol..

[43] Moraes Filho R.M., Jimenez H.J., Montarroyos A.V.V., Musser R.S., Silva M.M., Silva E.F., Martins L.S.S. (2011). Variabilidade genética em genótipos da coleção de germoplasma de *Citrus*, do Instituto Agronômico de Pernambuco Brejão-PE, por meio de marcadores moleculares ISSR. Citrus Res. Technol..

[44] Alzate-Marin A.L., Bonifacio-Anacleto F., de Moraes Filho R.M., Machado G.P., Nazareno A.G. (2016). Genetic Analysis across Life Stages of *Metrodorea nigra* (Rutaceae) in a Population Located in an Urban Landscape of Southeastern Brazil Using a New Set of Microsatellite Markers. Braz. J. Bot..

[45] Peakall R.O.D., Smouse P.E. (2012). GenAlEx 6.5: Genetic Analysis in Excel. Population Genetic Software for Teaching and Research—an Update. Bioinformatics.

[46] Hammer Ø., Harper D.A.T., Ryan P.D. (2001). PAST: Paleontological statistics software package for education and data analysis. Palaeontol. Electron..

[47] Segelbacher G., Cushman S.A., Epperson B.K., Fortin M.J., Francois O., Hardy O.J., Holderegger R., Taberlet P., Waits L.P., Manel S. (2010). Applications of landscape genetics in conservation biology: Concepts and challenges. Conserv. Genet..

[48] Smouse P.E., Peakall R.O.D. (1999). Spatial Autocorrelation Analysis of Individual Multiallele and Multilocus Genetic Structure. Heredity.

[49] Peakall R., Ruibal M., Lindenmayer D.B. (2003). Spatial Autocorrelation Analysis Offers New Insights into Gene Flow in the Australian Bush Rat, Rattus Fuscipes. Evolution.

[50] Ferreira-Ramos R., Monteiro M., Zucchi M.I., Pinheiro J.B., Martinez C.A., Mestriner M.A., Alzate-Marin A.L. (2011). Microsatellite markers for *Aspidosperma polyneuron* (Apocynaceae), an endangered tropical tree species. Am. J. Bot..

[51] Guidugli M.C., Nazareno A.G., Feres J.M., Contel E.P.B., Mestriner M.A., Alzate-Marin A.L. (2016). Small but not isolated: A population genetic survey of the tropical tree *Cariniana estrellensis* (Lecythidaceae) in a highly fragmented habitat. Heredity.

