# Supplementary material for: A Spatial Structure of Key Tree Species Metrodorea nigra St. Hill. (Rutaceae) Is Associated with Historical Disturbance and Isolation in Southeastern Brazil
Source: Plants (Basel). 2025 Feb 25;14(5):702. doi: 10.3390/plants14050702 (PMC11901653; doi:10.3390/plants14050702)
Supplement: Supplementary file 1 [file plants-14-00702-s001.zip › plants-3460819-supplementary.pdf]

Article

A Spatial Structure of Key Tree Species *Metrodorea nigra* St. Hill. (Rutaceae) Is Associated with Historical Disturbance and Isolation in Southeastern Brazil

Rômulo Maciel de Moraes Filho [1,2,*], Fernando Bonifácio-Anacleto [1,2], Fabio Alberto Alzate-Martinez [3], Carlos Alberto Martinez [4] and Ana Lilia Alzate-Marin [1,2,*]

[1] Plant Genetics Laboratory, Department of Genetics, Faculty of Medicine of Ribeirão Preto (FMRP-USP/RP), University of São Paulo, Ribeirão Preto 14049-900, Brazil; bonifacioanacleto@usp.br
[2] Graduate Program, Department of Genetics, Faculty of Medicine of Ribeirão Preto (FMRP-USP/RP), University of São Paulo, Av. Bandeirantes 3900, Ribeirão Preto 14049-900, Brazil
[3] Nawi Spatial Design and Research, Ribeirão Preto 14040-160, Brazil; nawistudio.contact@gmail.com
[4] Department of Biology, Ribeirão Preto School of Philosophy, Science and Literature (FFCLRP), University of São Paulo, Av. Bandeirantes 3900, Ribeirão Preto 14040-901, Brazil; carlosamh@ffclrp.usp.br
* Correspondence: romulommfilho@gmail.com (R.M.d.M.F.); anaalzate@fmrp.usp.br (A.L.A.-M.)

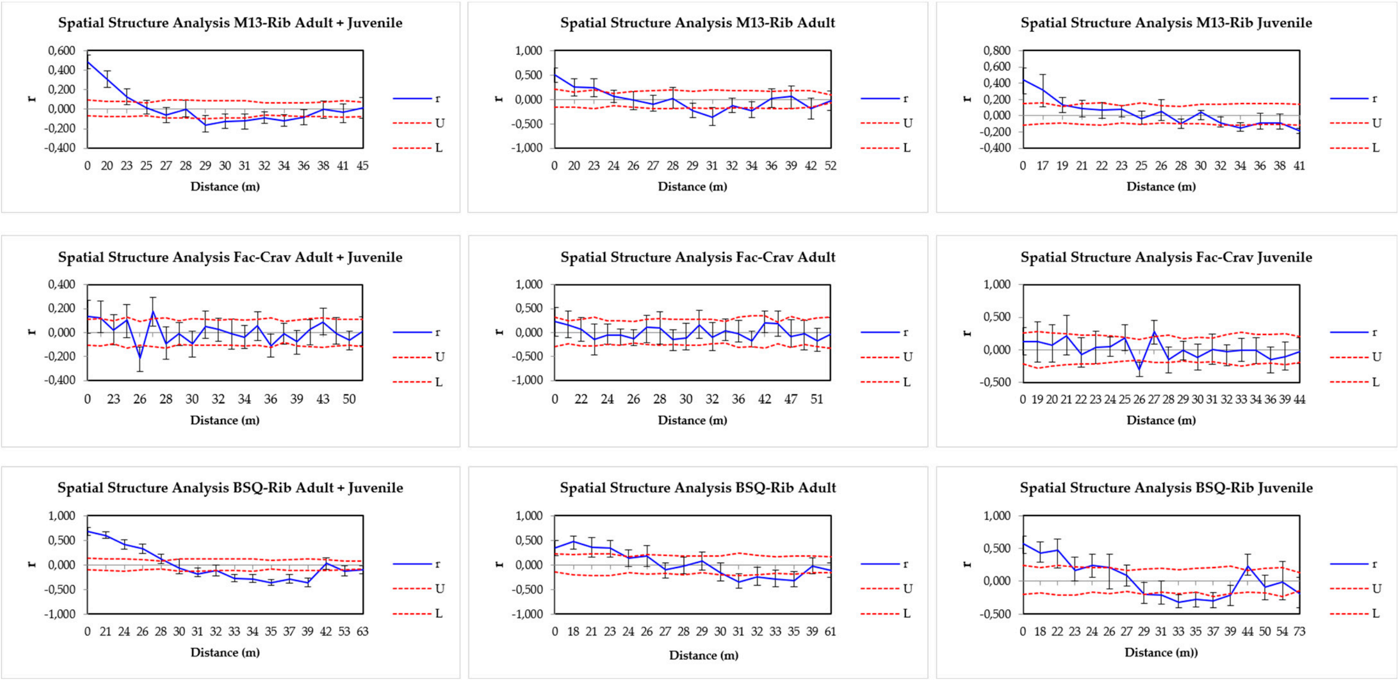

Figure S1. Spatial Autocorrelation Correlograms for M13-Rib, FAC-Crav, and BSQ-Rib from ISSR marker data considering adult and juvenile generations. The upper (U) and lower (L) red dashed lines represent the 95% confidence interval for $r = 0$, and the vertical bars indicate the 95% confidence interval for each r-value. Blue lines outside the confidence interval denote r-values significantly different from 0, indicating genetic structuring.

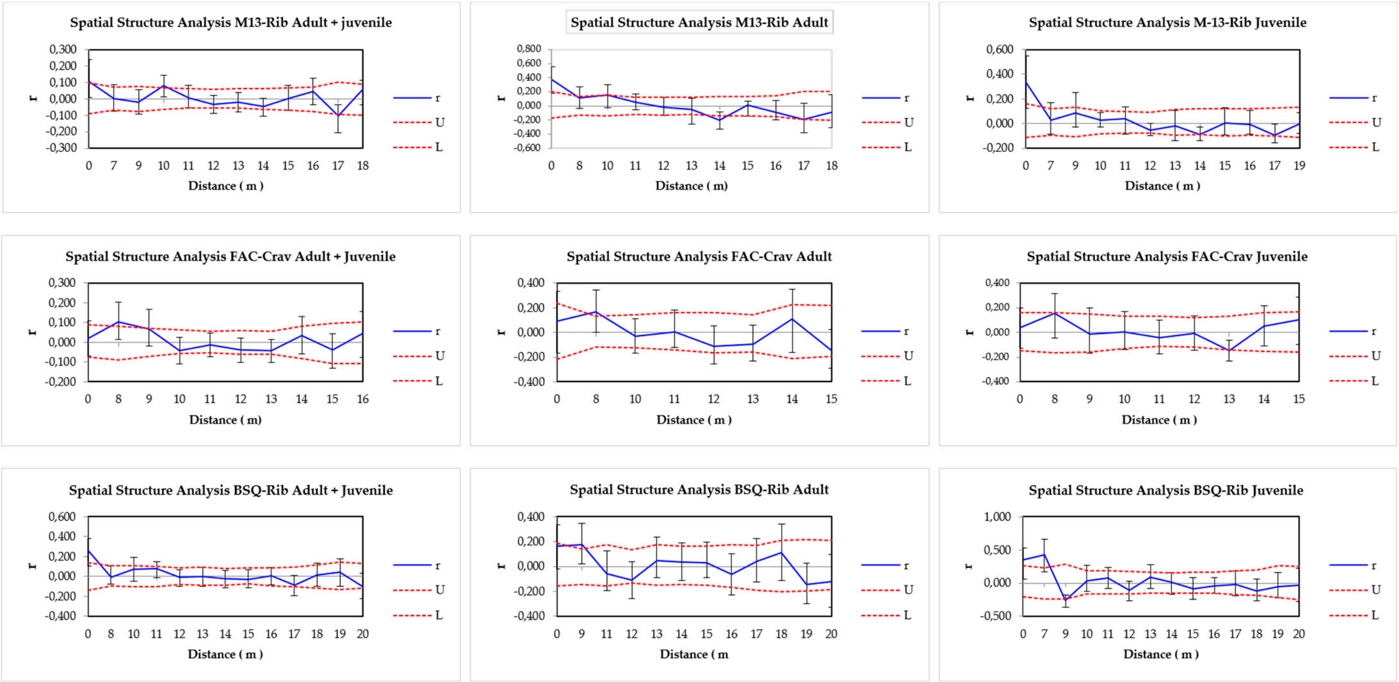

Figure S2. Spatial Autocorrelation Correlograms for M13-Rib, FAC-Crav, and BSQ-Rib from SSR marker data considering adult and juvenile generations. The upper (U) and lower (L) red dashed lines represent the 95% confidence interval for $r = 0$, and the vertical bars indicate the 95% confidence interval for each r-value. Blue lines outside the confidence interval denote r-values significantly different from 0, indicating genetic structuring.

Table S1. Polymorphism of ISSR primers used (Moraes Filho et al., 2015). PL: Polymorphic Loci; ML: Monomorphic Loci; $\hat{H}E$ = Nei genetic diversity. AT= Annealing Temperature

Primer		AT	N°Loci	PL	ML	$\hat{H}e$
UBC#1	ACACACACACACACACT	50	12	12	0	0.293
UBC#2	GAGAGAGAGAGAGAGAT	50	25	24	1	0.276
UBC#820	GTG TGT GTG TGT GTG TC	50	11	11	0	0.270
UBC#834	AGA GAG AGA GAG AGA GYT *	50	13	11	2	0.299
UBC#851	GTG TGT GTG TGT GTG TYG *	50	20	20	0	0.326
UBC#858	TGT GTG TGT GTG TGT GRT *	50	24	24	0	0.349
UBC#860	TGT GTG TGT GTG TGT GRA *	50	19	19	0	0.338
UBC#886	VDV CTC TCT CTC TCT CT *	50	12	12	0	0.338

* D = (A, G, T); R = (A, G); V = (A, C, G); Y = (C, T)

Table S2. Characteristics of the *M. nigra* microsatellite loci. Na = number of alleles; Ne = effective number of alleles; Ho = observed heterozygosity; He = expected heterozygosity; F = fixation index. AT= Annealing Temperature (Moraes Filho et al., 2015).

**Loci**		**Expected size**	**AT**	**Na**	**Ne**	**Ho**	**He**	**F**
Mtn1[1]	(TG)8	144-146	56	5	1.790	0.342	0.441	0.226
Mtn3[1]	(CA)10N8(CG)5(CA)6	273-283	50	18	9.380	0.658	0.893	0.263
Mtn13[1]	(GT)14	203-229	56	24	11.727	0.475	0.915	0.481
Mtn16[1]	(TG)3 CA(TG) 10	203-205	50	7	3.889	0.477	0.743	0.358
Mtn19[1]	(TA)3(TG)9(TA)3 (TG)10TT(TG)3	163-201	56	13	3.068	0.423	0.674	0.372
Mtn87[2]	(AGA)9	153–198	58	9	4.491	0.735	0.777	0.055
Mtn95[2]	(TG)18	210–275	62	28	22.043	0.503	0.955	0.473
Total				104	-	-	-	-
Mean				-	8.05	0.516	0.771	0.318

[1]Guidugli et al., (2012). [2]Alzate-Marin et al., 2016

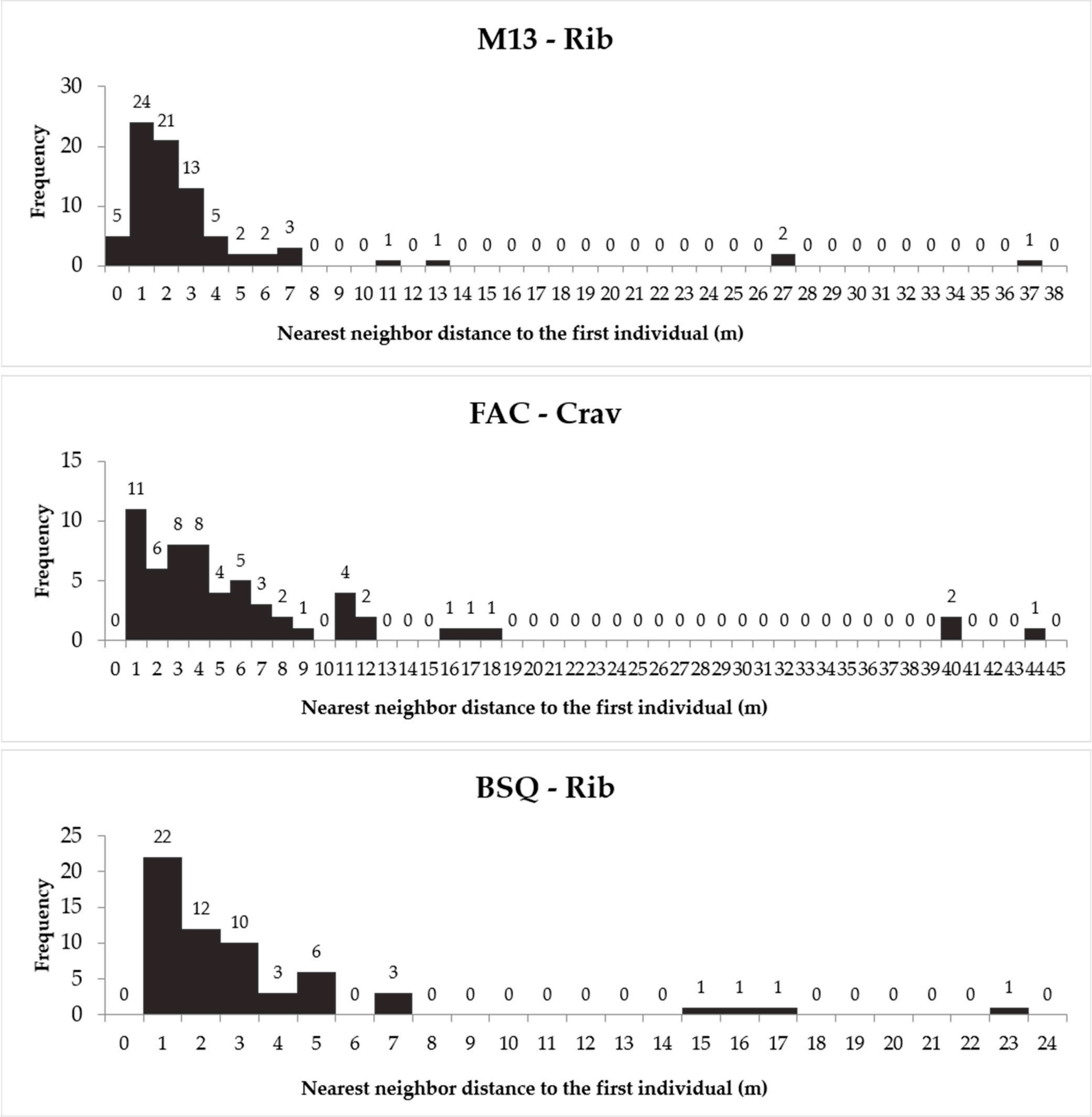

Figure S3. Frequency and distance to the nearest neighbor from the first individual for the M13-Rib, FAC-Crav, and BSQ-Rib populations.
